# Association of Traumatic Brain Injury With the Risk of Developing Chronic Cardiovascular, Endocrine, Neurological, and Psychiatric Disorders

**DOI:** 10.1001/jamanetworkopen.2022.9478

**Published:** 2022-04-28

**Authors:** Saef Izzy, Patrick M. Chen, Zabreen Tahir, Rachel Grashow, Farid Radmanesh, David J. Cote, Taha Yahya, Amar Dhand, Herman Taylor, Shirley L. Shih, Omar Albastaki, Craig Rovito, Samuel B. Snider, Michael Whalen, David M. Nathan, Karen K. Miller, Frank E. Speizer, Aaron Baggish, Marc G. Weisskopf, Ross Zafonte

**Affiliations:** 1 Divisions of Stroke, Cerebrovascular, and Critical Care Neurology, Department of Neurology, Brigham and Women’s Hospital, Boston, Massachusetts; 2 Harvard Medical School, Boston, Massachusetts; 3 Department of Environmental Health, Harvard T.H. Chan School of Public Health, Boston, Massachusetts; 4 The Football Players Health Study at Harvard University, Boston, Massachusetts; 5 Computational Neuroscience Outcomes Center, Department of Neurosurgery, Brigham and Women’s Hospital, Boston, Massachusetts; 6 Department of Neurosurgery, Keck School of Medicine of the University of Southern California, Los Angeles; 7 Network Science Institute, Northeastern University, Boston, Massachusetts; 8 Morehouse School of Medicine, Atlanta, Georgia; 9 Department of Physical Medicine and Rehabilitation, Massachusetts General Hospital, Brigham and Women’s Hospital, Boston; 10 Spaulding Rehabilitation Hospital, Charlestown, Massachusetts; 11 Department of Pediatrics, Massachusetts General Hospital, Boston; 12 Diabetes Center, Massachusetts General Hospital, Boston; 13 Neuroendocrine Unit, Massachusetts General Hospital, Boston; 14 Channing Division of Network Medicine, Department of Medicine, Brigham and Women’s Hospital, Boston, Massachusetts; 15 Department of Internal Medicine, Cardiovascular Performance Center, Massachusetts General Hospital, Boston

## Abstract

**Importance:**

Increased risk of neurological and psychiatric conditions after traumatic brain injury (TBI) is well-defined. However, cardiovascular and endocrine comorbidity risk after TBI in individuals without these comorbidities and associations with post-TBI mortality have received little attention.

**Objective:**

To assess the incidence of cardiovascular, endocrine, neurological, and psychiatric comorbidities in patients with mild TBI (mTBI) or moderate to severe TBI (msTBI) and analyze associations between post-TBI comorbidities and mortality.

**Design, Setting, and Participants:**

This prospective longitudinal cohort study used hospital-based patient registry data from a tertiary academic medical center to select patients without any prior clinical comorbidities who experienced TBI from 2000 to 2015. Using the same data registry, individuals without head injuries, the unexposed group, and without target comorbidities were selected and age-, sex-, and race-frequency–matched to TBI subgroups. Patients were followed-up for up to 10 years. Data were analyzed in 2021.

**Exposures:**

Mild or moderate to severe head trauma.

**Main Outcomes and Measures:**

Cardiovascular, endocrine, neurologic, and psychiatric conditions were defined based on *International Classification of Diseases, Ninth Revision* (*ICD-9*) or *International Statistical Classification of Diseases and Related Health Problems, Tenth Revision* (*ICD-10*). Associations between TBI and comorbidities, as well as associations between the comorbidities and mortality, were analyzed.

**Results:**

A total of 4351 patients with mTBI (median [IQR] age, 45 [29-57] years), 4351 patients with msTBI (median [IQR] age, 47 [30-58] years), and 4351 unexposed individuals (median [IQR] age, 46 [30-58] years) were included in analyses. In each group, 45% of participants were women. mTBI and msTBI were significantly associated with higher risks of cardiovascular, endocrine, neurologic, and psychiatric disorders compared with unexposed individuals. In particular, hypertension risk was increased in both mTBI (HR, 2.5; 95% CI, 2.1-2.9) and msTBI (HR, 2.4; 95% CI, 2.0-2.9) groups. Diabetes risk was increased in both mTBI (HR, 1.9; 95% CI, 1.4-2.7) and msTBI (HR, 1.9; 95% CI, 1.4-2.6) groups, and risk of ischemic stroke or transient ischemic attack was also increased in mTBI (HR, 2.2; 95% CI, 1.4-3.3) and msTBI (HR, 3.6; 95% CI, 2.4-5.3) groups. All comorbidities in the TBI subgroups emerged within a median (IQR) of 3.49 (1.76-5.96) years after injury. Risks for post-TBI comorbidities were also higher in patients aged 18 to 40 years compared with age-matched unexposed individuals: hypertension risk was increased in the mTBI (HR, 5.9; 95% CI, 3.9-9.1) and msTBI (HR, 3.9; 95% CI, 2.5-6.1) groups, while hyperlipidemia (HR, 2.3; 95% CI, 1.5-3.4) and diabetes (HR, 4.6; 95% CI, 2.1-9.9) were increased in the mTBI group. Individuals with msTBI, compared with unexposed patients, had higher risk of mortality (432 deaths [9.9%] vs 250 deaths [5.7%]; *P* < .001); postinjury hypertension (HR, 1.3; 95% CI, 1.1-1.7), coronary artery disease (HR, 2.2; 95% CI, 1.6-3.0), and adrenal insufficiency (HR, 6.2; 95% CI, 2.8-13.0) were also associated with higher mortality.

**Conclusions and Relevance:**

These findings suggest that TBI of any severity was associated with a higher risk of chronic cardiovascular, endocrine, and neurological comorbidities in patients without baseline diagnoses. Medical comorbidities were observed in relatively young patients with TBI. Comorbidities occurring after TBI were associated with higher mortality. These findings suggest the need for a targeted screening program for multisystem diseases after TBI, particularly chronic cardiometabolic diseases.

## Key Points

**Question:**

Is traumatic brain injury associated with long-term risk of cardiometabolic, neurological, or psychiatric comorbidities?

**Findings:**

In this cohort study including 4351 patients each with mild or moderate to severe TBI and 4351 frequency-matched unexposed patients without TBI, the rates of cardiovascular and endocrine comorbidities after TBI were significantly higher in patients with mild or moderate to severe TBI compared with patients without TBI. The risk of post-TBI comorbidities was higher in all age groups compared with age-matched unexposed patients, particularly in patients younger than 40 years, and post-TBI comorbidities were associated with higher mortality over a 10-year follow-up period.

**Meaning:**

These findings suggest that patients with TBI in all age groups may benefit from a proactive targeted screening program for chronic multisystem diseases, particularly cardiometabolic diseases, after injury.

## Teaser

This cohort study assesses incidence of comorbidities after traumatic brain injury compared with unexposed patients without head injury.

## Introduction

Traumatic brain injury (TBI) is a global health problem, with an estimated incidence of 64 to 74 million cases per year worldwide and is a leading cause of morbidity and mortality.^1,2^ Poor clinical outcomes may be due to the direct sequelae of TBI, pre-TBI comorbidities, or a combination of these factors. In addition, large-scale studies of American-style football players and military veterans have demonstrated that prior TBI is associated with multisystem chronic conditions, suggesting that recurrent severe head injuries may be associated with long-term health and functional status.^3,4^ The development of chronic medical comorbidities after TBI can complicate the course of recovery and increase health care costs and mortality.^5^ A number of registry-based studies have demonstrated increased risk of cardiovascular^5,6^ and metabolic disorders,^7^ as well as epilepsy,^8^ stroke,^6^ and depression,^9,10,11^ in the chronic phase of TBI recovery. However, most previous studies were based on self-report, focused on older age groups,^4,7^ or included patients with TBI and preexisting comorbidities,^5,7^ which precluded isolating the association of TBI severity with subsequent development of comorbidities and mortality, particularly in individuals who were otherwise healthy at the time of injury. In a 2021 study,^3^ we found higher risk for developing multisystem medical and behavioral comorbidities in previously healthy patients who sustained concussion. Notably, the risk of postconcussion comorbidities was higher in patients younger than 40 years old compared with age-matched unexposed patients.^3^ However, it is unclear whether our findings apply to more severe subtypes of TBI and whether post-TBI comorbidities are associated with mortality risk.^12,13^ A better understanding of the interplay between TBI and the development of medical and neurologic comorbidities can have important implications for preventive care, prognosis, and targeted screening in a high-risk population.

Here we present results from a large observational age-, race-, and sex-frequency–matched cohort study over a 10-year period to assess the risk and time to cardiovascular, endocrine, psychiatric, and neurological diagnoses in individuals with mild TBI (mTBI) and moderate to severe TBI (msTBI) compared with individuals without head injury, considered unexposed patients, and evaluate the association between post-TBI comorbidities and mortality after hospital discharge.

## Methods

This cohort study was approved by the Mass General Brigham (MGB) institutional review board, which waived the requirement for informed consent because data were deidenitfied. In this study, we used the MGB Research Patient Data Registry (RPDR), a web-based application that allows query of prospectively collected, centralized clinical data registry encompassing inpatient, outpatient, and emergency department diagnoses. The study was conducted following the Strengthening the Reporting of Observational Studies in Epidemiology (STROBE) reporting guideline for observational studies.

### Patient Selection

We included patients aged 18 years or older who had at least 1 inpatient or outpatient visit for mTBI or msTBI between 2000 and 2015, as well as a minimum of 1 inpatient or outpatient follow-up visit, from 6 months to up to 10 years after the incident TBI event between 2000 and 2019. The lag of at least 6 months was chosen deliberately to identify chronic comorbidities and exclude acute diagnoses. The unexposed group was identified using RPDR by selecting patients with the same age, sex, and race distribution as the TBI cohort. The same timeline criteria were used to select the unexposed group. The index date was the date of first TBI diagnosis for the TBI groups, and a random hospital or outpatient clinic encounter in the system for the unexposed group. Participants with a history of TBI and any comorbidity of interest before the index date were excluded. Demographic characteristics were determined based on the index date. Race and ethnicity are collected from medical records or self-reported by the patient, using standardized options for both race and ethnicity, including Black, Hispanic, White, other, or those with missing racial information; other was defined as Asian, Asian Pacific islander, Hawaiian, American Indian, and Middle Eastern, and those who selected multiple races or ethnicities. Patients with mTBI were frequency-matched on the demographic covariates (ie, age, sex, and race and ethnicity) with patients with msTBI, who were then frequency-matched for the same covariates with the unexposed group. The same unexposed group was used as the unexposed group for the mTBI and msTBI groups.

### Exposure

mTBI and msTBI were defined using the Centers of Disease Control and Prevention criteria and extracted using the validated *International Classification of Diseases, Ninth Revision* (*ICD-9*).^14,15^ The causes of TBI were defined based on the external cause of injury codes (*ICD-E*) using the ICD-PIC package in RStudio version 4.0.2 (R Project for Statistical Computing),^16^ which were categorized as due to fall, motor vehicle collision, struck by/against, assault, and other or unspecified. The latter includes pedestrian, pedal cyclist, transport other, drowning or submersion, other specified classifiable, other specified not elsewhere classifiable, and unspecified.

### Comorbidities

We studied 21 comorbidities across 4 organ systems. These included cardiovascular diseases and risk factors (ie, hypertension, hyperlipidemia, obesity, and coronary artery disease), endocrine disorders (ie, hypothyroidism, pituitary dysfunction, diabetes, adrenal insufficiency, and erectile dysfunction), neurological disorders (ie, ischemic stroke or transient ischemic attack, seizure, and all subtypes of dementia), and psychiatric disorders and substance misuse (ie, depression; bipolar disorder; psychosis; anxiety disorder; sleep disorder; suicide ideation, intent, or attempt; substance misuse; opioid misuse; and alcohol misuse). The diagnoses were determined using *ICD-9* or *International Statistical Classification of Diseases and Related Health Problems, Tenth Revision* (*ICD-10*) codes (eTable 1 in the Supplement).^17^ We assessed the risk and determined the time to development of each comorbidity after the index date based on the date when each comorbidity was first documented in the medical records.

### Statistical Analysis

Descriptive analyses used count and proportion or mean or median and IQR, as appropriate. Cox proportional hazards models, adjusted for age, sex, and race, were used to examine the associations of mTBI and msTBI with cardiovascular, endocrine, neurological, and psychiatric comorbidities. Patients were censored at the time of comorbidity diagnosis, death, or the last available encounter, whichever came first. The risk window extended from 6 months after the index encounter to 10 years after the initial encounter or to 2019, whichever came first. Schoenfeld residuals were calculated to test the proportional hazards assumption. In most instances, the proportional hazards assumption was satisfied. We used time-varying coefficients for TBI severity to address disproportionate hazards in 4 conditions: anxiety, hyperlipidemia, erectile dysfunction, and alcohol misuse.

In addition, a separate age-stratified analysis was performed. Kaplan-Meier plots were used to demonstrate the cumulative incidence rates of each comorbidity with log-rank test to compare the risk difference between case and unexposed group. Logistic regression was used to identify the association of each comorbidity with mortality, adjusting for age, sex, race, and TBI severity. Cox proportional hazards model were also adjusted for multiple comparisons using Bonferroni corrections.^18^ Interaction terms were used to explore the interaction of age and TBI severity in mortality.

To examine potential bias that might have been introduced by differences in the number of encounters before the comorbidity diagnosis, we compared median number of encounters before each diagnosis in the unexposed group vs mTBI and msTBI groups. In addition, we evaluated the risk of comorbidities diagnosed in patients with mTBI and msTBI that were diagnosed 1 year after the index date compared with the unexposed group. Significance was defined as *P* < .05 for all the analyses except *P* < .002 for the Bonferroni corrections in Cox proportional hazards models. All analyses were performed using RStudio version 4.0.2. Data were analyzed in 2021.

## Results

### Demographics

We identified a total of 49 000 patients with TBI between 2000 and 2015. Of these, we selected 4351 with mTBI (median [IQR] age, 45 [29-57] years) without prior comorbidities. These were age-, sex-, and race-frequency–matched from the same pool with patients diagnosed with msTBI who also had no prior comorbidities (median [IQR] age, 47 [30-58] years). Using the larger RPDR data pool, we selected an age-, sex-, and race-frequency–matched unexposed group without a diagnosis of TBI and similarly without comorbidities (median [IQR] age, 46 [30-58] years), who were registered between 2000 and 2015. In each group, 45% of participants were women. The most common mechanism of injury was fall, observed in 965 patients (22%) with mTBI and 1259 patients (29%) with msTBI. The median number of encounters among patients with TBI before the index date was similar to the unexposed group. However, patients with TBI had a higher number of total follow-up encounters after the index date (median [IQR]: mTBI, 10 [3-29] visits; msTBI: 10 [3-31] visits) compared with the unexposed group (median [IQR], 5 [2-14] visits) (Table 1).

**Table 1.** Baseline Patient Characteristics

Characteristic	No. (%)		
	Unexposed group (n = 4351)^a^	mTBI (n = 4351)^b^	msTBI (n = 4351)^b^
Sex			
Women	1955 (45)	1955 (45)	1955 (45)
Men	2396 (55)	2396 (55)	2396 (55)
Age, y			
Median (IQR)	46 (30-58)	45 (29-57)	47 (30-58)
18-40	1602 (37)	1602 (37)	1602 (37)
41-60	1861 (43)	1857 (43)	1857 (43)
>60	888 (21)	892 (21)	892 (21)
Race and ethnicity			
Black	316 (7)	284 (7)	239 (5)
Hispanic	255 (6)	256 (6)	280 (7)
White	3355 (77)	3216 (73)	3293 (76)
Other^c^	157 (4)	307 (7)	228 (5)
Missing	268 (6)	288 (7)	311 (7)
Mechanism of injury			
Fall	NA	965 (22)	1259 (29)
Motor vehicle collision	NA	920 (21)	1195 (27)
Struck by or against	NA	388 (9)	215 (5)
Other	NA	72 (2)	124 (3)
Unspecified	NA	2006 (46)	1558 (36)
Injury severity score, mean (SD)	NA	3.96 (0.3)	10.3 (5.1)
Encounters 1 y before index date, No.			
Mean (SD)	4 (5.5)	4 (6)	5 (8.5)
Median (IQR)	2 (1-4)	2 (1-5)	3 (1-6)
Encounters after the index date, No.			
Mean (SD)	13 (22)	24 (39)	26 (42)
Median (IQR)	5 (2-14)	10 (3-29)	10 (3-31)
Follow-up after the index date, mean (SD), y	3.9 (3.8)	4.4 (3.9)	5.6 (4.0)

Abbreviations: mTBI, mild traumatic brain injury; msTBI, moderate to severe traumatic brain injury; NA, not applicable.

^a^ Patients with msTBI were frequency-matched to patients without TBI from the same data pool.

^b^ Patients with mTBI were frequency-matched for age, sex, and race to patients with msTBI.

^c^ Other races included Asian, Asian Pacific Islander, Native Hawaiian, American Indian, and Middle Eastern individuals and those who reported more than 1 race.

### Multisystem Comorbidities After TBI

**Figure 1.**
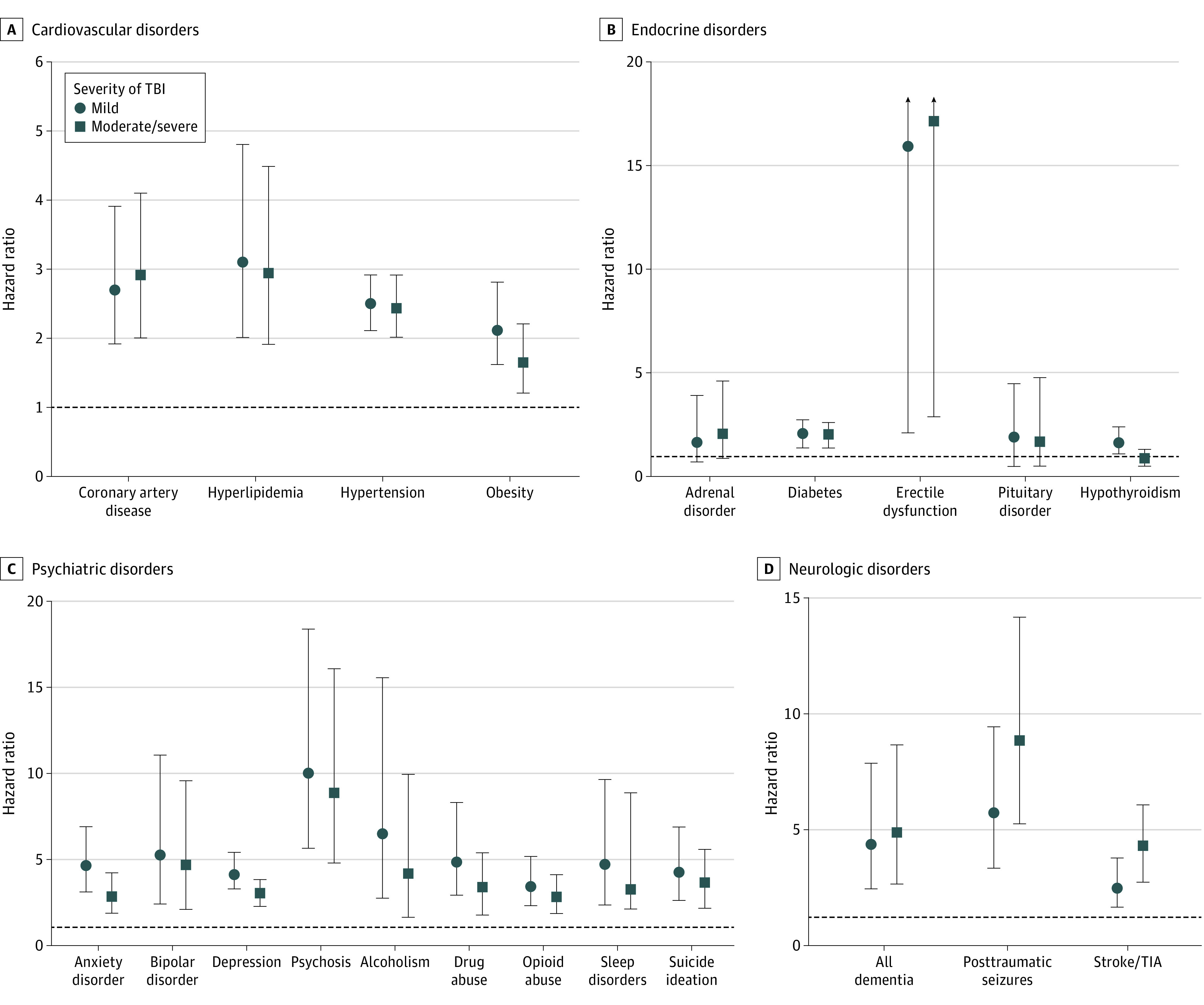
Risks of Multisystemic Comorbidities After Traumatic Brain Injury (TBI) Stratified by Severity Whiskers indicate 95% CIs; TIA, transient ischemic attack.

mTBI and msTBI were significantly associated with a higher risk of cardiovascular, endocrine, neurological, and psychiatric diseases. This included hypertension, with higher risk observed in patients with mTBI (HR, 2.5; 95% CI, 2.1-2.9) and patients with msTBI (HR, 2.4; 95% CI, 2.0-2.9). Similarly, the risk of hyperlipidemia, obesity, and coronary artery disease were higher in mTBI and msTBI groups compared with the unexposed group. TBI subgroups also had a higher risk of endocrine diseases, notably diabetes (mTBI: HR, 1.9; 95% CI, 1.4-2.7; msTBI: HR, 1.9; 95% CI, 1.4-2.6). Regardless of injury severity, patients with TBI had a higher risk of neurological and psychiatric diseases, particularly dementia and psychotic disorders, and risk of ischemic stroke or transient ischemic attack was also increased in mTBI (HR, 2.2; 95% CI, 1.4-3.3) and msTBI (HR, 3.6; 95% CI, 2.4-5.3) groups (Figure 1; eTable 2 in the Supplement).

Considering the higher number of total follow-up visits in the TBI groups, we conducted a sensitivity analysis to investigate whether more frequent follow-up was associated with higher diagnosis of comorbidities and potential encounter bias. We calculated the median number of encounters before the diagnosis of each comorbidity for mTBI, msTBI, and unexposed groups. In two-thirds of the outcomes assessed, no significant difference was found between the median number of medical encounters (eTable 3 in the Supplement). Restricting the analysis to comorbidities diagnosed after 1 year from the index encounter demonstrated similar results (eTable 4 in the Supplement).

### Age Stratification and Risk of Comorbidities

**Figure 2.**
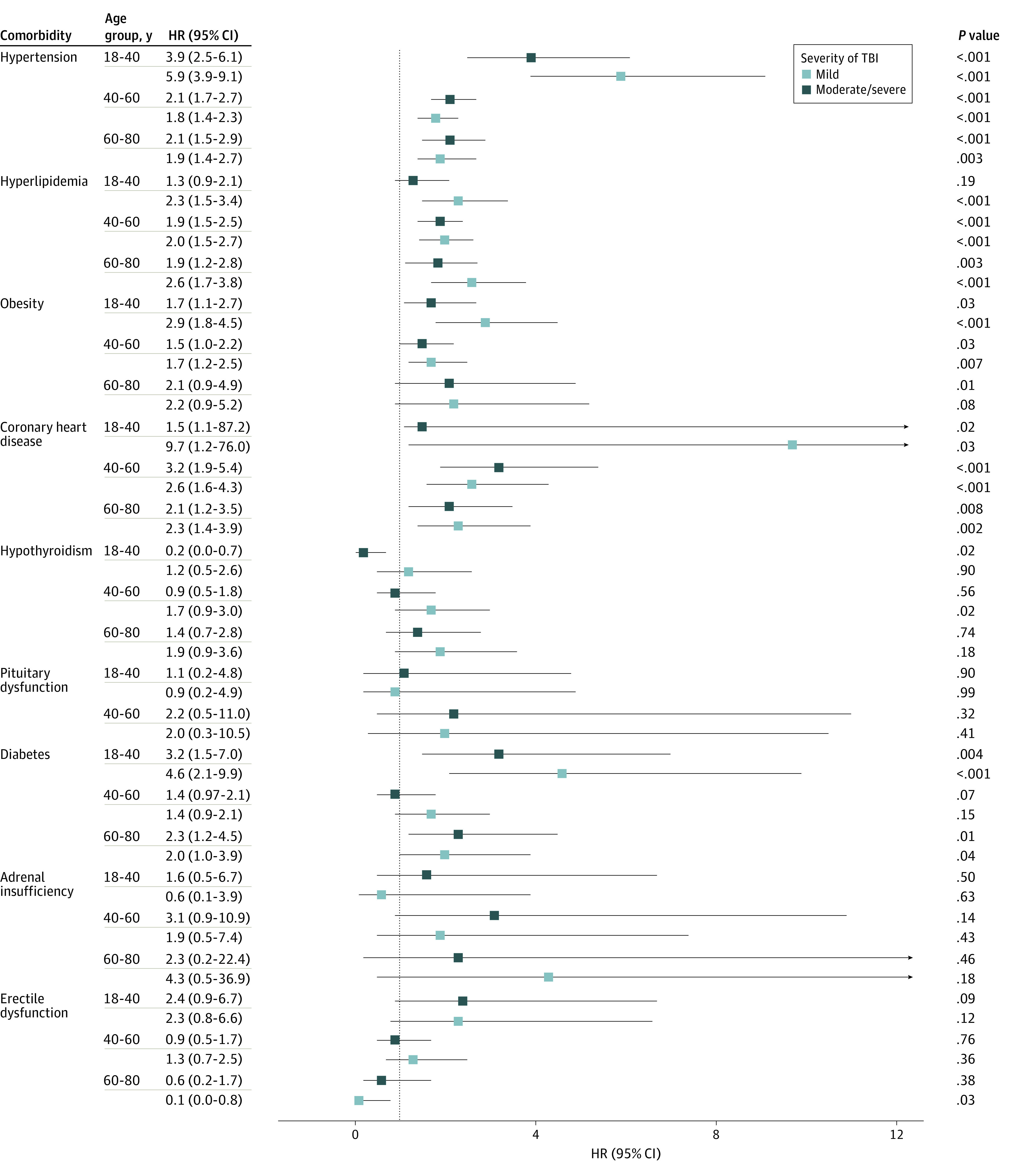
Risk of Cardiovascular and Endocrine Comorbidities After Traumatic Brain Injury (TBI) Stratified by Age Boxes indicate hazard ratios (HRs); whiskers, 95% CIs.

To determine whether the risk of developing comorbidities varied with age, we assessed the risks following TBI compared with age-matched unexposed patients by group for 3 age categories (18-40, 41-60, and >60 years). The younger age group (18-40 years) demonstrated significantly higher risk for cardiovascular diseases; in particular, hypertension risk was increased for both mTBI (HR, 5.9; 95% CI, 3.9-9.1) and msTBI (HR, 3.9; 95% CI, 2.5-6.1) groups, while hyperlipidemia (HR, 2.3; 95% CI, 1.5-3.4) and diabetes (HR, 4.6; 95% CI, 2.1-9.9) were increased in the mTBI group. We also found an increase in posttraumatic seizures, and psychiatric disorders in both mTBI and msTBI groups (Figure 2; eTable 5 in the Supplement). In the middle-aged (41-60 years) group, there was a higher risk of cardiovascular, psychiatric, and neurological disorders after mTBI and msTBI compared with the unexposed group. In this age group, patient with msTBI had higher risk of ischemic stroke and transient ischemic attach compared with the unexposed group. In patients older than 60 years, we found a higher risk of cardiovascular and neuropsychiatric comorbidities in both TBI subgroups compared with the unexposed group. In this age group, patients with mTBI also had a significant higher risk of anxiety disorder, while msTBI was associated with a higher risk of psychosis and seizures, compared with the unexposed group.

### Mortality Risk With TBI and Multisystem Comorbidities

Patients with msTBI, but not those with mTBI, were at higher risk of mortality compared with the unexposed group (432 deaths [9.9%] vs 250 deaths [5.7%]; *P* < .001) (eTable 6 in the Supplement). Comorbidities developing after TBI, in almost all disease domains, were associated with higher mortality, such as postinjury hypertension (HR, 1.34; 95% CI, 1.1-1.7), coronary artery disease (HR, 2.2; 95% CI, 1.6-3.0), adrenal insufficiency (HR, 6.2; 95% CI, 2.8-13.0), anxiety disorder (HR, 1.4; 95% CI, 1.1-1.9), substance misuse (HR, 3.7; 95% CI, 2.2-5.9), and dementia (HR, 3.0; 95% CI, 2.0-4.5) (Table 2). We did not find an interaction between age and TBI severity influencing mortality, suggesting an independent association for TBI severity (eTable 7 in the Supplement).

**Table 2.** Logistic Regression Analysis of Associations Between Post–Traumatic Brain Injury Comorbidities and Mortality

Comorbidities	Odds ratio (95% CI)
Cardiovascular disorders	
Hypertension	1.3 (1.1-1.7)
Hyperlipidemia	0.8 (0.6-1.1)
Obesity	0.4 (0.2-0.8)
Coronary heart disease	2.2 (1.6-3.0)
Endocrine disorders	
Hypothyroidism	0.5 (0.2-1.0)
Pituitary dysfunction	1.2 (0.1-6)
Diabetes	1.3 (0.8-1.9)
Adrenal insufficiency	6.2 (2.8-13.0)
Erectile dysfunction	0.5 (0.1-1.4)
Psychiatric disorders	
Depression	1.3 (0.9-1.8)
Bipolar disorder	2.0 (0.8-4.1)
Schizophrenia or psychosis	3.0 (2.1-4.4)
Anxiety disorder	1.4 (1.1-1.9)
Sleep disorder	1.1 (0.7-1.6)
Suicide ideation, intent, or attempt	2.4 (1.1-4.6)
Substance misuse	3.7 (2.2-5.9)
Opioid misuse	3.7 (2.0-6.0)
Alcohol misuse	2.5 (1.6-3.8)
Neurological disorders	
Ischemic stroke or transient ischemic attack	1.6 (1.1-2.4)
Seizure disorder	3.4 (2.3-4.8)
Dementia	3.0 (2.0-4.5)

### Time to Development of Comorbidities Following TBI

**Figure 3.**
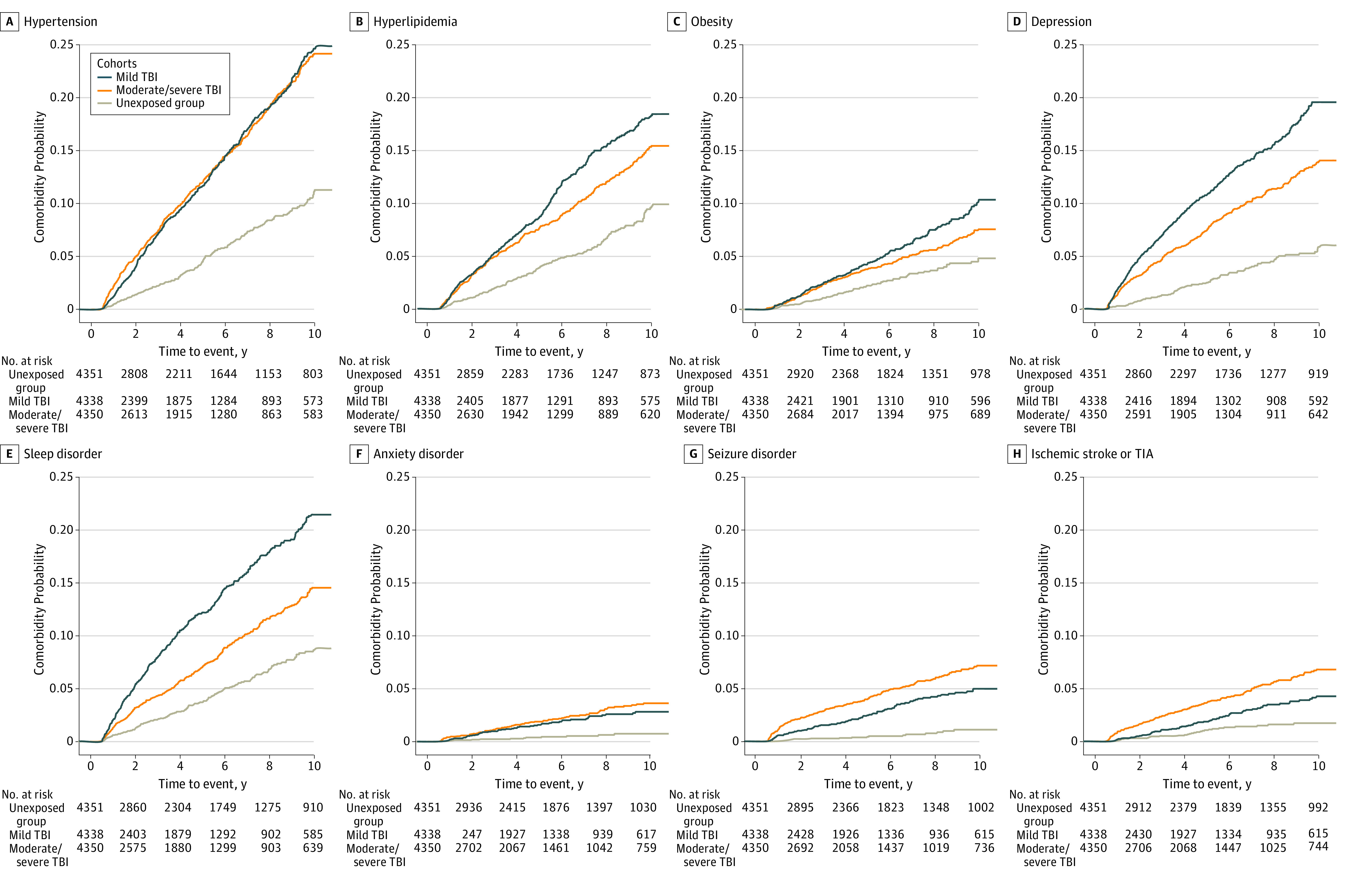
Kaplan-Meier Graphs of Risk of Multisystem Comorbidities Developing Post–Traumatic Brain Injury (TBI) Including TIA indicates transient ischemic attack.

Comorbidities across all systems emerged within a median (IQR) of 3.49 (1.76-5.96) years after both mTBI and msTBI and, generally, occurred with less latency than was seen in the unexposed group (eTable 8 in the Supplement). The cumulative incidence risk for hypertension, hyperlipidemia, obesity, depression, anxiety, seizures, and ischemic stroke or transient ischemic attack was significantly higher after mTBI and msTBI compared with unexposed group (Figure 3).

## Discussion

This cohort study assessed the risk of multisystem comorbidities after mTBI and msTBI compared with age-, sex-, and race-frequency–matched unexposed patients without head injuries over a 10-year follow-up period in individuals without prior comorbidities. To our knowledge, the risks of incident comorbidities in previously healthy patients who sustained mTBI or msTBI have not been previously reported. We demonstrated a higher risk of multiple conditions across cardiovascular, endocrine, neurological, and psychiatric systems even in the young age group of 18 to 40 years.

A few prior registry-based studies have reported increased risk of cardiovascular disorders in the chronic phase of TBI recovery. However, these studies were either self-reported or cross-sectional, leaving it unknown whether these comorbidities began before or after the brain injury.^5,6^ Our results suggest an association between TBI of varied severity and cardiovascular diseases in all age groups without baseline diagnoses. Possible explanations include behavioral and lifestyle changes, including physical inactivity, unhealthy diet, social isolation, systemic metabolic changes, or increased propensity for other risk diseases, including sleep disorders and depression.^9,19,20,21^ Other possibilities include certain biologic substrates secondary to TBI and the alteration in environmental factors after the injury that may lead to the emergence of a cluster of comorbidities driving one another and ultimately setting the scene for the clinical outcomes observed in this study.^22^ Recent clinical and experimental studies suggest TBI may alter systemic metabolomic, gut flora, and immune pathways.^23,24,25^ Therefore, the higher risk of comorbidities after TBI likely represents a combination of direct (hormonal and inflammatory changes caused by injury) and indirect factors (psychosocial risk factors).

We found, as previously reported, higher risk of psychiatric comorbidities independent of injury severity.^5,26^ The directionality of psychiatric conditions and TBI is challenging to determine, and this represents 1 of the limitations of this study. A recent study showed a higher use of analgesics and psychotropic medication before and after TBI.^27^ Substance abuse, lifestyle, genetic, and environmental factors all may play substantial roles in the etiology of psychiatric disorders.^22,27^ It is possible that patients with TBI had undiagnosed or undocumented psychiatric conditions that might have put them at higher risk for TBI but were not revealed until after TBI.

Several comorbidities that developed after TBI are more prevalent in older individuals. However, age stratification demonstrated that the younger age groups were also at high risk. Similarly, higher diabetes risk after TBI followed a bimodal distribution, sparing the middle-age subgroup. There was a higher risk of psychotic disorders in the middle-aged and older subgroups, replicating the results of prior studies.^14^ As was shown previously, the risk of dementia is higher in older individuals with any TBI severity.^14,28^ This suggests a subacute-chronic disease state rather than simply higher diagnostic scrutiny or an immediate complication of acute brain injury.

TBI is associated with higher mortality after hospital discharge, with the risk factors including injury severity and preexisting comorbidities.^29^ We found that post-TBI cardiovascular disorders, as well as psychiatric and neurologic disorders, were associated with mortality after adjustment for baseline demographic characteristics and TBI severity. It is possible that the neuropsychiatric diseases may be the primary substrate for the cardiometabolic comorbidities that in turn increased mortality risk. The directionality and causality of comorbidities and their association with long-term mortality require further investigation.

### Limitations

This study has several limitations. By using an institutional registry database that relies on accurate and comprehensive documentation of *ICD-9* and *ICD-10* codes, there is the possibility of information bias. The patient population evaluated is from a tertiary academic system in the Northeast United States, which may limit generalizability. The possibility that comorbidities reflect short-term transient outcomes from TBI cannot be ruled out. However, we investigated this possibility by limiting the analyses to comorbidities beginning 6 months after the initial encounter and by the sensitivity analyses including only the comorbidities beginning 1 year after injury. We acknowledge the possibility of encounter bias, given that patients with TBI had a higher number of encounters and longer follow-up duration. We calculated the median number of encounters for TBI subgroups and unexposed group before comorbidities getting diagnosed. In two-thirds of the outcomes assessed, no significant differences were found in analyses stratified by median number of medical encounters; however, there may still be unmeasured encounter (or ascertainment) bias. We did not address how comorbidities interact with each other. Additionally, there may be other confounders, including socioeconomic status, personality disorders, or undocumented comorbidities, that were present before injury, influencing the risk of post-TBI morbidities and mortality.

## Conclusions

This cohort study found that patients with mTBI and msTBI were at increased risk of developing long-term cardiovascular, endocrine, psychiatric, and neurological comorbidities. The risk of post-TBI comorbidities was higher in all age groups compared with an age-, sex-, and race-frequency–matched unexposed group, and notably so in patients younger than 40 years. Comorbidities after TBI were associated with higher mortality. These findings suggest a need for proactive screening of chronic systemic diseases after brain injury of any severity.

## Notes

Supplement. **eTable 1.** *ICD-9* and *ICD-10* Codes Used for Determining Diagnosis of Comorbidities **eTable 2.** Hazard Ratios for Multisystem Comorbidities Developing After TBI **eTable 3.** Median Number of Encounters Before Diagnosis of Comorbidities in TBI and Unexposed Groups **eTable 4.** Hazard Ratios for Multisystem Comorbidities Developing After Mild and Moderate or Severe TBI Compared With Unexposed Group After 1 Year From Index Encounter **eTable 5.** Hazard Ratio of Comorbidities Developing After TBI Stratified by Age **eTable 6.** Association Between TBI Severity and Long-term Mortality **eTable 7.** Interaction Analysis Between Moderate or Severe TBI and Age **eTable 8.** Time to Develop Comorbidities After Index Date or TBI Diagnosis
