# Supplementary material for: Association of Traumatic Brain Injury With the Risk of Developing Chronic Cardiovascular, Endocrine, Neurological, and Psychiatric Disorders
Source: JAMA Netw Open. 2022 Apr 28;5(4):e229478. doi: 10.1001/jamanetworkopen.2022.9478 (PMC9051987; doi:10.1001/jamanetworkopen.2022.9478)
Supplement: Supplement. — eTable 1. ICD-9 and ICD-10 Codes Used for Determining Diagnosis of Comorbidities eTable 2. Hazard Ratios for Multisystem Comorbidities Developing After TBI eTable 3. Median Number of Encounters Before Diagnosis of Comorbidities in TBI and Unexposed Groups eTable 4. Hazard Ratios for Multisystem Comorbidities Developing After Mild and Moderate or Severe TBI Compared With Unexposed Group After 1 Year From Index Encounter eTable 5. Hazard Ratio of Comorbidities Developing After TBI Stratified by Age eTable 6. Association Between TBI Severity and Long-term Mortality eTable 7. Interaction Analysis Between Moderate or Severe TBI and Age eTable 8. Time to Develop Comorbidities After Index Date or TBI Diagnosis [file jamanetwopen-e229478-s001.pdf]

Supplemental Online Content

Izzy S, Chen PM, Tahir Z, et al. Association of traumatic brain injury with the risk of developing chronic cardiovascular, endocrine, neurological, and psychiatric disorders. *JAMA Netw Open*. 2022;5(4):e229478. doi:10.1001/jamanetworkopen.2022.9478

eTable 1. *ICD-9* and *ICD-10* Codes Used for Determining Diagnosis of Comorbidities
eTable 2. Hazard Ratios for Multisystem Comorbidities Developing After TBI
eTable 3. Median Number of Encounters Before Diagnosis of Comorbidities in TBI and Unexposed Groups
eTable 4. Hazard Ratios for Multisystem Comorbidities Developing After Mild and Moderate or Severe TBI Compared With Unexposed Group After 1 Year From Index Encounter
eTable 5. Hazard Ratio of Comorbidities Developing After TBI Stratified by Age
eTable 6. Association Between TBI Severity and Long-term Mortality
eTable 7. Interaction Analysis Between Moderate or Severe TBI and Age
eTable 8. Time to Develop Comorbidities After Index Date or TBI Diagnosis

This supplemental material has been provided by the authors to give readers additional information about their work.

© 2022 Izzy S et al. *JAMA Network Open.*

eTable 1. *ICD-9* and *ICD-10* Codes Used for Determining Diagnosis of Comorbidities

Disorder	ICD-9	ICD-10
Cardiovascular Disorders		
Hypertension	401, 402, 403, 404, 405	I10, I11, I12, I13, I15, I16
Hyperlipidemia	272	E78
Obesity	278	E66, Z68.25-Z68.44
Coronary artery disease	410, 411, 412, 413, 414	I21, I22, I23, I24, I25
Endocrine disorders		
Hypothyroidism	244	E03
Pituitary dysfunction	253	E23
Diabetes mellitus	250, 790.29, 790.21	E08, E09, E11, E13
Adrenal insufficiency	255.4-255.9	E27.0-E27.7
Erectile dysfunction	607.84	N52, F52.21
Psychiatric disorders		
Depression	296.2, 296.3, 296.8, 300.4, 311	F32, F33
Bipolar disorder	296.4, 296.5, 296.6, 296.7	F25, F31
Schizophrenia/ psychosis	295, 296.89, 296.9, 298	F06, F20, F22, F23, F28, F29
Anxiety disorder	300, 300.2, 308, 309	F41, F40.8, F40.9, F43.2
Sleep disorder	307.4, 327, 780.5, 347	F51, G47
Suicide ideation/intent/attempt	E950 - E958, V62.84	R45. 851, X83, T40, Z91.5
Substance misuse	304.1-304.6, 304.8-304.9, 305.2, 305.3, 305.4, 305.6-305.9	F12, F13, F14, F15, F16, F18, F19
Opioid misuse	304.0, 304.7, 305.5	F11
Alcohol misuse	303, 305.0	F10
Neurological disorders		
Ischemic stroke/TIA	433, 434, 435, 436, 437	I63, I65, I66, I67
Dementia	290.0-290.4, 290.8, 290.9, 294.1, 294.2, 331	F01, F02, F03, G30, G31
Seizure disorder	780.33, 780.39, 345	R56.1, R56.9, G40

© 2022 Izzy S et al. *JAMA Network Open.*

eTable 2. Hazard Ratios for Multisystem Comorbidities Developing After TBI

	Unexposed group		Mild TBI			Moderate/severe TBI		
**Comorbidities**	**Number**	**Follow-up (person-years)**	**Number**	**Follow-up (person-years)**	**HR (95% CI)**	**Number**	**Follow-up (person-years)**	**HR (95% CI)**
Cardiovascular disorders								
Hypertension	172	16366	454	17098	2.5 (2.1-2.9) *	530	19800	2.4 (2.0-2.9) *
Hyperlipidemia	145	16401	343	17341	3.1 (2.0-4.8) *	333	20518	2.9 (1.9-4.5) *
Obesity	76	16564	175	18027	2.1 (1.6-2.8) *	162	21315	1.6 (1.2-2.2) *
Coronary artery disease	39	16721	117	18314	2.7 (1.9-3.9) *	156	21397	2.9 (2.0-4.1) *
Endocrine disorders								
Hypothyroidism	42	16720	76	18585	1.7 (1.1-2.4)	50	21907	0.9 (0.5-1.3)
Pituitary dysfunction	6	16788	9	18678	1.6 (0.5-4.8)	11	21949	1.6 (0.5-4.8)
Diabetes mellitus	60	16639	129	18185	1.9 (1.4-2.7) *	149	21360	1.9 (1.4-2.6) *
Adrenal insufficiency	7	16778	14	18629	1.7 (0.7-4.4)	22	21891	2.4 (0.9-5.5)
Erectile dysfunction	33	16800	41	18558	15.9 (2.1-121)	48	21786	17.1 (2.9-123)
Psychiatric disorders								
Depression	94	16538	384	17262	4.1 (3.3-5.3) *	315	20631	2.9 (2.3-3.7) *
Bipolar disorder	10	16782	45	18531	5.2 (2.4-11) *	47	21818	4.5 (2.1-9.5) *
Psychosis	13	16750	133	18164	10 (5.6-18.3) *	135	21411	8.7 (4.8-16) *
Anxiety disorder	140	16445	428	17171	4.6 (3.1-6.8) *	316	20723	2.8 (1.9-4.2) *
Sleep disorder	62	16800	227	17866	4.7 (2.3-9.6) *	182	21240	4.4 (2.1-8.8) *
Suicide ideation/intent/attempt	10	16785	47	18542	4.2 (2.6-6.8) *	53	21818	3.5 (2.2-5.5) *
Substance misuse	22	16741	98	18317	4.8 (2.9-8.2) *	97	21586	3.2 (1.8-5.3) *
Opioid misuse	17	16762	87	18390	3.4 (2.3-5.1) *	70	21710	2.7 (1.9-4.1) *
Alcohol misuse	34	16746	119	18258	6.4 (2.7-15.5) *	116	21558	4.0 (1.6-9.9) *
Neurological disorders								
Ischemic stroke/TIA	31	16759	76	18442	2.2 (1.4-3.3) *	153	21359	3.6 (2.4-5.3) *
Seizure disorder	17	16765	94	18353	5.0 (2.9-8.4) *	167	21267	7.6 (4.6-13) *
Dementia	13	16776	56	18517	3.8 (2.1-6.9) *	81	21694	4.2 (2.3-7.6) *
**Adjusted for age, sex, and race**<br>**Significance denoted by * if p< 0.002 (Bonferroni Adjustment)**<br>**Reference= Unexposed group**								

© 2022 Izzy S et al. *JAMA Network Open.*

eTable 3. Median Number of Encounters Before Diagnosis of Comorbidities in TBI and Unexposed Groups

**Comorbidity**	**Median number of encounters before the diagnosis of comorbidity**		
	**Unexposed group**	**Mild TBI**	**Moderate/severe TBI**
Cardiovascular disorders			
Hypertension	7 (3-19)	7 (3-19)	9 (3-25) *
Hyperlipidemia	10 (4-20)	9 (3-23)	13 (5-33) *
Coronary heart disease	9 (4-26)	14 (6-32)	15 (6-37)
Obesity	8 (4-18)	11 (4-23)	12 (3-42)
Endocrine disorders			
Hypothyroidism	10 (5-26)	18 (6-45) *	17 (7-46) *
Pituitary dysfunction	3 (2-13)	30 (13-89)	18 (15-73)
Diabetes mellitus	10 (3-19)	11 (4-38) *	12 (4-30) *
Adrenal insufficiency	9 (3-33)	21 (8-37)	17 (9-53)
Erectile dysfunction	12 (5-25)	11 (4-29)	15 (7-32)
Psychiatric disorders			
Depression	12 (4-22)	9 (3-25)	10 (4-26)
Bipolar disorder	2 (1.5-21)	13 (5-34)	20 (8-36)
Schizophrenia/ psychosis	9 (3-12)	10 (3-39) *	14 (4-39) *
Anxiety disorder	13 (4-25)	10 (4-24)	12 (5-30)
Suicide ideation/intent/attempt	26 (9-42)	9 (5-34)	15 (8-35)
Substance misuse	10 (4-28)	8 (3-18)	9 (3-27)
Opioid misuse	4 (2-9)	10 (3-36) *	12 (5-31) *
Alcohol misuse	7 (4-17)	8 (3-17)	8 (3-19)
Neurological disorders			
Ischemic Stroke/TIA	16 (3-22)	11 (3-39) *	11 (4-28)
Seizure disorder	10 (5-31)	11 (4-29)	9 (3-26)
Dementia	8 (6-11.5)	12 (4.5-26)	12 (3-23)
**Significance denoted by * < 0.05**<br>**Reference= Unexposed group, t-test**			

© 2022 Izzy S et al. *JAMA Network Open.*

eTable 4. Hazard Ratios for Multisystem Comorbidities Developing After Mild and Moderate or Severe TBI Compared With Unexposed Group After 1 Year From Index Encounter

Comorbidities	Unexposed group		Mild TBI			Moderate/severe TBI		
	Number	Follow-up (person-years)	Number	Follow-up (person-years)	HR (95% CI)	Number	Follow-up (person-years)	HR (95% CI)
Cardiovascular disorders								
Hypertension	159	16422	418	17280	2.5 (2.0-2.9) *	460	20241	2.2 (1.8-2.6) *
Hyperlipidemia	133	16465	307	17553	2.2 (1.8-2.7) *	298	20750	1.7 (1.4-2.1) *
Obesity	69	16608	164	18087	2.2 (1.7-2.9) *	149	21385	1.7 (1.3-2.2) *
Coronary artery disease	34	16729	112	18341	3.0 (2.1-4.4) *	143	21477	3.1 (2.4-4.3) *
Endocrine disorders								
Hypothyroidism	40	16726	74	18590	1.7 (1.1-2.5)	48	21911	0.9 (0.6-1.3)
Pituitary dysfunction	6	16788	9	18678	1.6 (0.5-4.8)	9	21955	1.3 (0.4-3.9)
Diabetes mellitus	58	16648	119	18238	1.8 (1.3-2.5) *	140	21420	1.7 (1.2-2.4) *
Adrenal insufficiency	6	16778	13	18634	1.8 (0.7-4.9)	19	21908	2.3 (0.9-5.9)
Erectile dysfunction	33	16800	36	18581	0.9 (0.6-1.6)	41	21829	0.8 (0.6-1.5)
Psychiatric disorders								
Depression	86	16565	328	17553	3.9 (3.0-4.9) *	270	20944	2.6 (2.1-3.4) *
Bipolar disorder	8	16788	39	18560	5.1 (2.3-11.5) *	37	21871	3.9 (1.7-8.8) *
Psychosis	12	16759	113	18260	9.3 (5.0-17.4) *	117	21523	8 (4.3-14.9) *
Anxiety disorder	124	16517	370	17484	2.9 (2.4-3.6) *	276	20998	1.8 (1.5-2.2) *
Sleep disorders	55	16652	194	18045	3.1 (2.3-4.2) *	156	21394	2.1 (1.5-2.9) *
Suicide ideation/intent/attempt	9	16785	42	18563	4.7 (2.2-10.0) *	49	21833	4.5 (2.1-9.5) *
Substance misuse	19	16746	86	18372	4.3 (2.6-7.0) *	84	21657	3.4 (2.1-5.6) *
Opioid misuse	17	16762	79	18421	4.4 (2.6-7.4) *	62	21766	2.8 (1.6-4.7) *
Alcohol misuse	32	16747	109	18310	3.4 (2.2-5.0) *	106	21598	2.6 (1.8-3.9) *
Neurological disorders								
Ischemic Stroke/TIA	28	16760	71	18459	2.2 (1.4-3.4) *	124	21528	3.1 (2.1-4.7) *
Seizure disorder	15	16769	77	18430	4.6 (2.7-8.0) *	130	21513	6.5 (3.8-11) *
Dementia	10	16790	50	18550	4.4 (2.2-8.7) *	67	21781	4.4 (2.3-8.5) *
**Adjusted for age, sex, and race<br>Significance denoted by * if p< 0.002 (Bonferroni Adjustment)<br>Reference= Unexposed group**								

© 2022 Izzy S et al. *JAMA Network Open.*

eTable 5. Hazard Ratio of Comorbidities Developing After TBI Stratified by Age

**Comorbidities**	**18-40 years**		**41-60 years**		**>60 years**	
	**Mild TBI**	**Moderate TBI**	**Mild TBI**	**Moderate TBI**	**Mild TBI**	**Moderate TBI**
	**HR (95% CI)**	**HR (95% CI)**	**HR (95% CI)**	**HR (95% CI)**	**HR (95% CI)**	**HR (95% CI)**
Cardiovascular disorders						
Hypertension	5.9 (3.9-9.1) *	3.9 (2.5-6.1) *	1.8 (1.4-2.3) *	2.1 (1.7-2.7) *	1.9 (1.4-2.7) *	2.1 (1.5-2.9) *
Hyperlipidemia	2.3 (1.5-3.4) *	1.3 (0.9-2.1)	2.0 (1.5-2.7) *	1.9 (1.5-2.5) *	2.6 (1.7-3.8) *	1.9 (1.2-2.8)
Obesity	2.9 (1.8-4.5) *	1.7 (1.1-2.7)	1.7 (1.2-2.5)	1.5 (1.0-2.2)	2.2 (0.9-5.2)	2.1 (0.9-4.9)
Coronary heart disease	9.7 (1.2-76.0)	1.5 (1.1-87.2)	2.6 (1.6-4.3) *	3.2 (1.9-5.4) *	2.3 (1.4-3.9) *	2.1 (1.2-3.5)
Endocrine disorders						
Hypothyroidism	1.2 (0.5-2.6)	0.2 (0.03-0.7)	1.7 (0.9-3.0)	0.9 (0.5-1.8)	1.9 (0.9-3.6)	1.4 (0.7-2.8)
Pituitary dysfunction	0.9 (0.2-4.9)	1.1 (0.2-4.8)	2.0 (0.3-10.5)	2.2 (0.5-11.0)	N/A	N/A
Diabetes mellitus	4.6 (2.1-9.9) *	3.2 (1.5-7.0)	1.4 (0.9-2.1)	1.4 (0.97-2.1)	2.0 (1.0-3.9)	2.3 (1.2-4.5)
Adrenal insufficiency	0.6 (0.1-3.9)	1.6 (0.5-6.7)	1.9 (0.5-7.4)	3.1 (0.9-10.9)	4.3 (0.5-36.9)	2.3 (0.2-22.4)
Erectile dysfunction	2.3 (0.8-6.6)	2.4 (0.9-6.7)	1.3 (0.7-2.5)	0.9 (0.5-1.7)	0.1 (0.01-0.8)	0.6 (0.2-1.7)
Psychiatric disorders						
Depression	3.3 (2.3-4.5) *	2.3 (1.7-3.3) *	4.9 (3.5-7.2) *	3.6 (2.5-5.2) *	6.1 (3-12.7) *	2.7 (1.2-5.9) *
Bipolar disorder	2.8 (1.1-7.2)	3.7 (1.5-9.0)	9.4 (2.2-40.0)	5.5 (1.3-23.8)	N/A	N/A
Schizophrenia/ psychosis	5.2 (2.5-1067) *	4.8 (2.4-9.9) *	2.7 (6.7-112) *	2.5 (6-100) *	2.2 (2.9-161)	1.4 (1.8-103.7) *
Anxiety disorder	2.6 (1.9-3.4) *	1.5 (1.2-2.1)	3.6 (2.6-5.0) *	2.5 (1.8-3.5) *	3.6 (2.1-6.4) *	1.8 (0.9-3.2)
Suicide ideation/intent/attempt	2.7 (1.2-6.1)	3.2 (1.5-7.0)	1.8 (2.4-135)	1.5 (1.9-107.8)	N/A	N/A
Substance misuse	3.5 (2.1-5.9) *	2.6 (1.5-4.4) *	7.2 (2.5-20.3) *	7.2 (2.6-20) *	N/A	N/A
Opioid misuse	4.6 (2.3-9.1) *	2.9 (1.5-5.9)	4.8 (2.1-10.7) *	3.2 (1.4-7.3)	N/A	N/A
Alcohol misuse	2.3 (1.4-3.9) *	1.7 (1.0-2.9)	4.8 (2.5-9.2) *	4.1 (2.2-7.8) *	N/A	N/A
Neurological disorders						
Ischemic Stroke/TIA	0.4 (0.1-2.0)	4.4 (1.7-11.5)	1.8 (0.9-3.6)	3.6 (1.9-6.5) *	2.9 (1.6-5.6) *	3.7 (2.0-6.7) *
Seizure disorder	6.8 (2.7-17.5) *	8.9 (3.5-22.3) *	3.6 (1.8-7.3) *	6.3 (3-12.3) *	7.4 (1.7-31.8)	10.8 (2.6-45.5) *
Dementia	2 (0.2-22)	7.9 (0.9-63)	4.3 (0.9-19.7)	7.0 (1.6-30.2)	3.6 (1.8-7.1) *	3.9 (2.0-7.9) *
**Adjusted for sex and race**<br>**Significance denoted by * if p< 0.002 (Bonferroni Adjustment)**<br>**Reference= Unexposed group**						

© 2022 Izzy S et al. *JAMA Network Open.*

eTable 6. Association Between TBI Severity and Long-term Mortality

	Unexposed group	Mild TBI	p-value	Moderate-severe TBI	p-value
**Total deaths**	250 (5.7%)	237 (5.4%)	0.54	432 (9.9%)	<0.001
**Age at death (years), median**	75 (58 – 86)	75 (57 – 86)	0.89	71 (55 – 85)	0.07
**Time to death (years), median**	3.0 (1.3 – 5.1)	4.1 (1.9 – 5.9)	0.01	3.3 (1.8 – 6.0)	0.01
**Time to death (years), mean**	3.5 (±2.5)	4.1 (±2.5)		4.0 (±2.6)	
**Adjusted for age, sex, and race**<br>**Reference = Unexposed group**					

© 2022 Izzy S et al. *JAMA Network Open.*

eTable 7. Interaction Analysis Between Moderate or Severe TBI and Age

**Outcome**	**Age 41-60**	**Age >60**	**Moderate/severe TBI**	**Interaction (mod/sevTBI * Age 41-60)**	**Interaction (mod/sev TBI * Age > 60)**
**Mortality**	3.8 (2.2 – 6.9)<br>p-value = **4.8e-6**	22.1 (13.4 – 39.5)<br>p-value = **<2e-16**	2.1 (1.1 – 3.9)<br>p-value = **0.02**	1.23 (0.6 – 2.4)<br>p-value = 0.558	0.86 (0.4 – 1.6)<br>p-value = 0.659
Reference= 18-40 age group with mild TBI					

© 2022 Izzy S et al. *JAMA Network Open.*

eTable 8. Time to Develop Comorbidities After Index Date or TBI Diagnosis

**Comorbidities**	**Median time to comorbidities (IQR)**		
	**Unexposed group**	**Mild TBI cohort**	**Moderate/ severe TBI cohort**
Cardiovascular disorders			
Hypertension	4.5 (2.3-6.4)	3.4 (1.9-5.9) *	3.5 (1.6-6.2) *
Hyperlipidemia	4.3 (2.3-7.1)	3.5 (1.7-5.7) *	3.5 (1.8-6.3) *
Obesity	4.0 (2.3-6.1)	4.1 (2.2-6.6)	3.4 (2.1-6.3)
Coronary heart disease	3.7 (1.7-6.8)	4.3 (2.4-6.3)	3.7 (1.9-7.1)
Endocrine disorders			
Hypothyroidism	4.8 (2.9-7.2)	4.6 (2.5-6.1)	5.2 (2.9-8.2)
Pituitary dysfunction	3.6 (2.6-4.3)	4.3 (3.3-7.9)	3.4 (1.9-6.7)
Diabetes mellitus	4.4 (2.7-6.9)	3.9 (2.4-6.1)	3.7 (2.1-6.3)
Adrenal insufficiency	3.9 (2.1-5.2)	2.4 (2.1-4.5)	3.4 (1.9-5.1)
Erectile dysfunction	8.4 (4.3-10)	4.0 (1.9-6.4) *	3.9 (1.5-5.9) *
Psychiatric disorders			
Depression	3.8 (2.2-6.2)	2.9 (1.4-5.3) *	3.2 (1.4-5.7)
Bipolar disorder	3.9 (2.2-5.9)	3.5 (1.9-5.9)	5.5 (2.8-7.7)
Schizophrenia/ psychosis	3.7 (1.9-4.4)	2.9 (1.6-5.9)	3.3 (1.5-5.9)
Anxiety disorder	4.2 (2.1-6.2)	2.9 (1.4-5.4) *	3.8 (1.8-6.1)
Suicide ideation/intent/attempt	4.8 (2.5-5.8)	3.3 (2.2-5.3)	4.9 (2.5-6.6)
Substance misuse	4.2 (1.9-4.9)	3.2 (1.6-6.3)	3.4 (1.5-5.9)
Opioid misuse	4.6 (2.7-6.9)	3.9 (1.9-6.1)	4.8 (2.4-7.2)
Alcohol misuse	4.9 (3.2-7.4)	3.2 (1.6-5.5) *	4.4 (2.3-6.2)
Neurological disorders			
Ischemic Stroke/TIA	4.1 (1.9-4.9)	4.1 (2.4-5.9)	3.2 (1.4-5.9)
Seizure disorder	4.3 (1.5-6.7)	3.7 (1.5-5.7)	2.7 (1.0-5.5)
Dementia	3.7 (1.0-5.1)	3.1 (1.8-5.2)	3,5 (1.9-6.1)
**Reference= unexposed group, t-test performed.**<br>**Significance denoted by * < 0.05**			

© 2022 Izzy S et al. *JAMA Network Open.*
